# Somatic Mutations in Exocrine Pancreatic Tumors: Association with Patient Survival

**DOI:** 10.1371/journal.pone.0060870

**Published:** 2013-04-02

**Authors:** P. Sivaramakrishna Rachakonda, Andrea S. Bauer, Huaping Xie, Daniele Campa, Cosmeri Rizzato, Federico Canzian, Stefania Beghelli, William Greenhalf, Eithne Costello, Michaela Schanne, Anette Heller, Aldo Scarpa, John P. Neoptolemos, Jens Werner, Markus Büchler, Jörg D. Hoheisel, Kari Hemminki, Nathalia Giese, Rajiv Kumar

**Affiliations:** 1 Division of Molecular Genetic Epidemiology, German Cancer Research Center, Heidelberg, Germany; 2 Division of Functional Genome Analysis, German Cancer Research Center, Heidelberg, Germany; 3 Department of Gastroenterology, Tongji Hospital, Tongji Medical College, Huazhong University of Science and Technology, Wuhan, China; 4 Department of Pathology and Diagnostics, Università di Verona, Verona, Italy; 5 National Institute for Health Research, Pancreas Biomedical Research Unit and Cancer Research UK Centre, Liverpool, United Kingdom; 6 Department of General Surgery, University Hospital Heidelberg, Heidelberg, Germany; 7 Center for Primary Health Care Research, Lund University, Malmö, Sweden; University of Nebraska Medical Center, United States of America

## Abstract

*KRAS* mutations are major factors involved in initiation and maintenance of pancreatic tumors. The impact of different mutations on patient survival has not been clearly defined. We screened tumors from 171 pancreatic cancer patients for mutations in *KRAS* and *CDKN2A* genes. Mutations in *KRAS* were detected in 134 tumors, with 131 in codon 12 and only 3 in codon 61. The GGT>GAT (G12D) was the most frequent mutation and was present in 60% (80/134). Deletions and mutations in *CDKN2A* were detected in 43 tumors. Analysis showed that *KRAS* mutations were associated with reduced patient survival in both malignant exocrine and ductal adenocarcinomas (PDAC). Patients with PDACs that had *KRAS* mutations showed a median survival of 17 months compared to 30 months for those without mutations (log-rank P = 0.07) with a multivariate hazard ratio (HR) of 2.19 (95%CI 1.09–4.42). The patients with G12D mutation showed a median survival of 16 months (log-rank-test P = 0.03) and an associated multivariate HR 2.42 (95%CI 1.14–2.67). Although, the association of survival in PDAC patients with *CDKN2A* aberrations in tumors was not statistically significant, the sub-group of patients with concomitant *KRAS* mutations and *CDKN2A* alterations in tumors were associated with a median survival of 13.5 months compared to 22 months without mutation (log-rank-test P = 0.02) and a corresponding HR of 3.07 (95%CI 1.33–7.10). Our results are indicative of an association between mutational status and survival in PDAC patients, which if confirmed in subsequent studies can have potential clinical application.

## Introduction

Pancreatic ductal adenocarcinoma (PDAC) is the most fatal form of pancreatic malignancy with a 5 year survival of less than 4% [1], [2]. Tumor heterogeneity, lack of early detection methods and refractoriness to conventional chemotherapy all contribute to the poor outcome [2]. Surgical resection has limited potential for cure, with less than 20% of patients eligible for surgery with curative intent, due to local spread or metastasis [3]. PDAC is thought to develop from PanIN lesions (pancreatic intraepithelial neoplasia) through progressive accumulation of somatic alterations in critical genes [4], [5]. Despite a repertoire of information, studies linking somatic alterations in PDAC with patient survival are lacking.

Over the years somatic mutations have been shown to be legitimate targets for anti-cancer drugs because of casual relationship with tumor formation and maintenance [6]. Histological indistinct tumors, based on the mutational profiles are reported to be differentially amenable to chemotherapeutics [7]. Specific chemotherapeutics, based on mutational status, in colorectal, lung, melanoma and other cancer types are already part of cancer treatments [8]–[12]. Despite *KRAS* being the most frequently mutated oncogene in pancreatic cancer with a reported frequency ranging between 20 and 100%, it has not been so far utilized in categorization of tumors for clinical purposes [13]. Though, some previous reports have suggested association of *KRAS* mutations in resected pancreatic cancers with prognosis [14], [15].

Most of the earlier reports on *KRAS* mutations in pancreatic cancer were based on relatively small tumor numbers that lacked statistical power to determine association with the disease outcome. In order to address the issue of frequency of *KRAS* mutation in pancreatic cancer and impact of those mutations on disease outcome, we have in this study included a series of fully characterized 171 pancreatic tumors with complete patient data.

## Results

The 163 patients with malignant tumors in this study comprised the following: i) 143 ductal adenocarcinomas that also included 5 adenosquamous and 4 anaplastic undifferentiated variants, ii) 16 rare carcinomas that were comprised of 2 acinar cell carcinomas, 2 (microcystic) tubulo-papillary carcinomas, 9 intraductal papillary mucinous neoplasm (IPMN, invasive type), 2 solid pseudopapillary neoplasms (Frantz tumors) and 1 cystadenocarcinoma, and iii) 4 papillary (ampulla of Vater) carcinomas. The non-malignant group was composed of 4 benign lesions in the form of serous cystic adenomas (SCA) and premalignant lesions in the form of 1 mucinous cystic neoplasm (MCN) and 3 non-invasive IPMN (Table 1 and Table S3). All patients except nine received standard Gemcitabine treatment. Out of remaining nine patients, eight received 5-fluorouracil/folinic acid and one patient received 5-fluorouracil and interferon-alpha together with radiation therapy (Table S3).

**Table 1:** Clinical-pathological parameters of pancreatic cancer patients.

		Total (N = 171)	Census status (N = 159)		
		Number (%)	Number (censored)	Median survival Months (95% CI)	Log-rank *P* *
**All categories**		159 (93)	159 (45)	16 (9–26)	
		12 (6 no follow up; 6 deaths due to other causes)			
**Gender**	Male	100 (58)	92 (27)	19 (16–28)	0.19
	Female	71 (42)	67 (18)	17 (11–22)	
**Age at surgery (years)**	Median** = ** 65 (56–70); Mean = 63 ± 11.31	171	159 (45)	16 (9–26)	–
**Histologic variants**		*n = 171*	*n = 159 (45)*		
benign	Serous cystadenoma, SCA	4 (2)	4 (4)	–	–
premalignant	Mucinous cystic neoplasm, MCN	1 (1)	1 (1)	–	–
	Intraductal papillary mucinous neoplasm, IPMN (low grade)	3 (2)	1 (1)	–	–
malignant	**Ductal adenocarcinomas**	*n = 143*	*n = 135 (26)*		
	PDAC	134 (78)	128 (26)	17 (13–22)	–
	Adenosquamous carcinoma	5 (3)	4 (0)	13 (3–17)	–
	Anaplastic undifferentiated carcinoma	4 (2)	3 (0)	3 (2–4)	–
	**Carcinomas: rare cases**	*n = 16*	*n = 15 (11)*		
	Acinar cell carcinoma	2 (1)	2 (2)	–	–
	Microcystic tubulopapillary adenocarcinoma	2 (1)	2 (1)	–	–
	Intraductal papillary mucinous neoplasm, IPMN (invasive carcinoma)	9 (5)	8 (5)	39 (6 –n.c†)	–
	SPN/Frantz's tumor	2 (1)	2 (2)	–	–
	Cystadenocarcinoma	1 (1)	1 (1)	–	–
ampullary region	**Carcinoma of ampulla Vateri**	4 (2)	3 (2)	–	–
**Tumor location**	Pancreatic head	111 (66)	108 (23)	17 (13–22)	0.79
	Pancreatic body	19 (11)	15 (4)	22 (5–32)	
	Pancreatic tail	20 (11)	19 (8)	19 (8–n.c†)	
	Overlapping sites	13 (8)	11 (6)	14 (6–n.c†)	
	Ampulla Vateri	4 (2)	3 (2)	–	
**TNM status**	Tis (T0)	3 (2)	3 (3)	.	0.12
	T1	3 (2)	3 (2)	.	
	T2	2 (1)	2 (0)	30.5 (26–35)	
	T3	130 (76)	122 (28)	18 (14–22)	
	T4	19 (11)	17 (4)	12 (9–19)	
	no status	14 (8)	12 (4)		
	N0	31 (18)	30 (12)	22 (14–32)	0.32
	N1	128 (75)	119 (27)	17 (13–22)	
	no status	12 (7)	10 (6)		
	M0	141 (82)	134 (36)	18 (14–23)	0.25
	M1	18 (11)	15 (3)	14.5 (9–27)	
	no status	12 (7)	10 (6)		
**Grade**	G1	7 (4)	6 (1)	24 (5–44)	< 0.0001
	G2	88 (51)	85 (20)	19 (16–24)	
	G3	57 (33)	53 (13)	13 (9–19)	
	no status	15 (9)	13 (11)		
	Anaplastic type	4 (2)	3 (0)	2.5 (2–3)	

* Logrank *P*-value for the differences in survival.

† Median survival upper limit not calculable due to insufficient number of events.

Mutation detection for *KRAS* gene was standardized using DNA from cell lines with known *KRAS* mutation. The sensitivity of SSCP, determined by titration experiments, showed that point mutations in tumor samples up to 5% tumor content were detectable. This provided confidence that our inclusion of tumor samples, only if those had at least 10% tumor content (n = 171), would more than adequately enable the detection of mutations. Another criterion applied for mutation detection was reproducibility. Mutations were scored only when band shifts were reproducible in at least two independent experiments. Repeat experiments using SSCP followed by DNA sequencing were used for confirmation and identification of mutations (Figure S2). We also obtained independent confirmation of *KRAS* mutations in a random sub-set (n = 6) analyzed blindly in the reference laboratory of the Institute of Pathology, University Hospital of Heidelberg.

In the *KRAS* gene, we detected 134 mutations in 171 tumors (78%), with 131 mutations in exon 2 and 3 mutations in exon 3 (Table 1). Mutations in exon 2 in all tumors were localized to codon 12. Out of 131 tumors that carried mutation at codon 12, 61% tumors had GGT>GAT (G12D, 80 of 131) mutation, followed by GGT>CGT (G12R, 23 of 131, 18%), GGT>GTT (G12V, 22 of 131, 17%), GGT>TGT (G12C, 4 of 131, 3%), GGT>GCT (G12A, 1 of 131) and GGT>GTC (G12V, 1 of 131). Three tumors carried mutations in exon 3 that were confined to codon 61 featuring the Q61H mutation due to CAA>CAC base change. The mutation frequency in ductal adenocarcinomas was 82% (117 of 143) including adenosquamous and anaplastic undifferentiated tumors. All 4 of the ampulla of Vater tumors showed *KRAS* mutation, while 7 of 9 IPMN-malignant types harbored mutation (Table 1 and Table S3).

A total of 43 tumors (25%) showed aberrations in the *CDKN2A* gene. Of the *CDKN2A* alterations in 43 tumors, 9 carried point mutations and the remainder showed deletion at the locus. All the point mutations in the gene were located in exon 2. Two tumors carried mutation at codon 80 (CGA>TGA, R80*), 3 at codon 83 (CAC>TAC, H83Y), followed by solitary tumors with mutations at codon 58 (CGA>TGA, R58*), codon 129 (TAC>TAA, Y129*), codon 130 (CTG>CAG, L130Q) and one tumor had 2 base pair insertion of GG at codon 78 (CTC>CGGTC). Deletions at the 9p21 locus were detected with varying frequency with 17–20% in the *CDKN2A* (p16^INK4a^) and 26–28% within the promoter associated with exon 1β of p14^ARF^ transcript.

Univariate analyses showed that among clinico-pathological factors, only tumor grade significantly affected overall survival in the studied cohort (Table 1). Presence of *KRAS* mutations tended to shorten survival of patients in general (n = 150; P = 0.07) and in all studied sub-categories (except tumor stage T4), however without reaching statistical significance (Table S2). In 150 patients with malignant exocrine tumors, the activating *KRAS* mutations were associated with reduction in median survival time nearly by half (17 vs 30 months, Kaplan-Meier method with log-rank test P = 0.07; Figure S3A). The presence of *KRAS* mutations was associated with poor survival in tumor stage III (HR = 1.94, P = 0.03; Table S2). Risk factors such as smoking, alcohol consumption or diabetes had no effect on patient survival either with or without *KRAS* mutations. A multivariate Cox regression model that included age, gender, TNM, tumor grade and tumor histology as co-variants confirmed *KRAS* mutational status as a potential independent prognostic marker with a hazard ratio (HR) of 1.87 (95%CI 0.99–3.51, P = 0.05; Table 2). Analysis with specific types of *KRAS* mutations at codon 12 showed that the G12D variant was associated with a median survival time of 16 months compared to 30 months for wildtype *KRAS* (log-rank test, P = 0.02; Figure S3B) and HR of 1.99 (95%CI 1.02–3.90, P = 0.05; multivariate cox-regression analysis; Table 2). Patients with any *CDKN2A* aberration in tumors showed a shorter median survival time of 13.5 months compared to 19 months in patients without aberrations, however, the difference was not statistically significant (log-rank P = 0.14). The corresponding HR was 1.55 (95%CI 0.97–2.48, P = 0.07; Table 2). The survival of patients with concomitant *KRAS* mutations and *CDKN2A* aberrations (n = 31) was poorest with median survival time of 13 months compared to 30 months for patients without any mutations in either *KRAS* or *CDKN2A* (log rank P = 0.03; Figure S3C). Out of 31 tumors with concomitant mutations in *KRAS* and *CDKN2A*, 30 were stage III or IV. Twenty three of those tumors were lymphnode positive. The HR for the presence of concomitant aberrations in both genes was 2.77 (95%CI 1.23–6.23, P = 0.01; Table 2).

**Table 2:** Multivariate Cox regression analysis for the effect of mutations on survival in malignant exocrine cancer patients.

Parameter	Total	Alive (censored)	Median Survival Months (95% CI)	*P*	Hazard ratio (HR)*	95% CI
***KRAS*** **Wt**	31	13	30 (13–44)		1.00 (reference)	
***KRAS*** **mutants**	119	24	17 (13–21)	0.05	1.87	0.99–3.51
***KRAS*** **: G12D (GAT)**	70	12	16 (11–23)	0.05	1.99	1.02–3.90
***KRAS*** **: G12R (CGT)**	22	5	18 (13–31)	0.93	1.04	0.39–2.75
***KRAS*** **: G12V (GTT/GTC)**	20	5	16 (11–19)	0.09	2.27	0.90–5.82
***KRAS*** **: Q61H (CAC)**	3	0	6 (4–35)	0.01	59.56	2.79–1272.33
***K-ras*** **: others**	3	2	–	0.02	231.44	2.27–23560.74
***CDKN2A*** **Wt**	112	30	19 (16–24)		1.00 (reference)	
***CDKN2A*** **mutants**	38	7	13.5 (9–18)	0.07	1.55	0.97–2.48
***KRAS + CDKN2A*** **wt**	24	11	30 (13–44)		1.00 (reference)	
***KRAS + CDKN2A*** **mutants**	31	5	13(7–18)	0.01	2.77	1.23–6.23

* Hazard ratio and corresponding P-value for effect of mutations on survival calculated after adjusting with gender, age, TNM status, tumor differentiation grade and histology.

**Figure 1:**
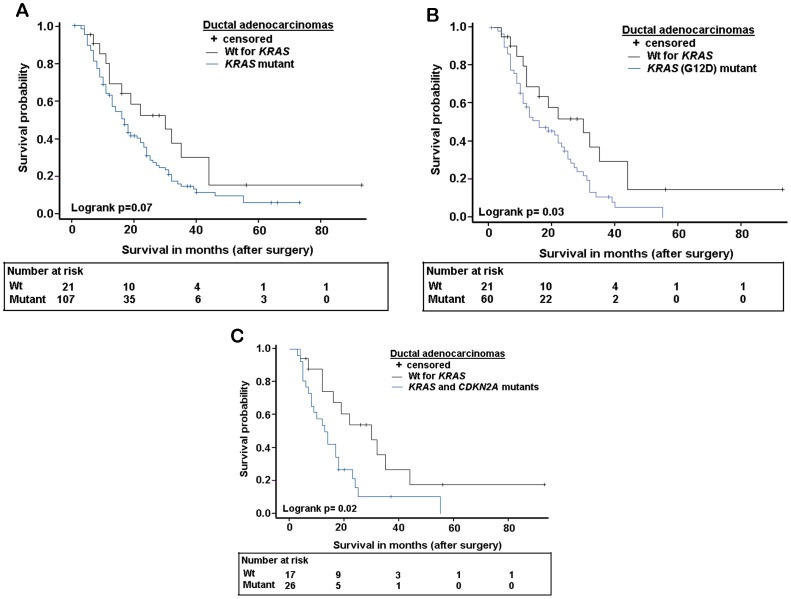
Kaplan-Meier survival curves showing difference in overall survival in PDAC patients with and without mutations. (A) Median survival of patients with any *KRAS* mutations was 17 months against 30 months for patients without mutations in the gene. (B) Median survival of patients with *KRAS* codon 12 GGT>GAT (G12D) mutations was 16 months against 30 months for patients without any mutation in *KRAS*. (C) Median survival of patients with concomitant alterations in *KRAS* and *CDKN2A* genes was 13.5 months against 22 months for patients without any alterations in both *KRAS* and *CDKN2A*.

Analysis of survival only for PDAC patients (n = 128) showed a similar association with *KRAS* mutational status. Patients with *KRAS* mutations were associated with a median survival time of 17 months compared to 30 months for those without mutations (log-rank test P = 0.07; Figure 1A). Multivariate Cox regression showed association of *KRAS* mutations with a HR of 2.19 (95%CI 1.09–4.42; Table 3). PDAC patients with G12D mutation in *KRAS* had 16 months of median survival (log rank P = 0.03; Figure 1B). And the sub-group of PDAC patients with concomitant mutations in *KRAS* and *CDKN2A* had a shortest survival of 13.5 months (log rank test P = 0.02; Figure 1C) and a HR of 3.07 (95%CI 1.33–7.10; Table 3).

**Table 3:** Multivariate Cox regression analysis for the effect of mutations on survival in PDAC patients.

Parameter	Total	Alive (censored)	Median Survival Months (95% CI)	*P*	Hazard ratio (HR)*	95% CI
***KRAS* Wt**	21	7	30 (12–44)		1.00 (reference)	
***KRAS* mutants**	107	19	17 (13–21)	0.03	2.19	1.09–4.42
***KRAS* G12D (GAT)**	60	9	16 (11–23)	0.02	2.42	1.14–2.67
***KRAS*: G12R (CGT)**	22	5	18 (13–31)	0.94	1.04	0.39–2.73
***KRAS*: G12V (GTT/GTC)**	20	4	16 (8–19)	0.08	2.30	2.36–992.70
***KRAS*: Q61H (CAC)**	3	0	6 (4–35)	0.01	48.43	0.70–544.44
***K-ras*: others**	2	1	17 (–)	0.03	126.13	1.42–11221.60
***CDKN2A* Wt**	98	22	19 (14–24)		1.00 (reference)	
***CDKN2A* mutants**	30	4	13.5 (9–18)	0.06	1.60	0.99–2.60
***KRAS + CDKN2A* wt**	17	6	22 (12–35)		1.00 (reference)	
***KRAS + CDKN2A* mutants**	26	3	13.5 (8–18)	0.01	3.07	1.33–7.10

* Hazard ratio and corresponding P-value for effect of mutations on survival calculated after adjusting with gender, age, TNM status, and tumor differentiation grade.

## Discussion

Mutations in *KRAS* and *CDKN2A* genes in pancreatic cancer are well documented; however, their influence on disease outcome in patients with exocrine pancreatic tumors has remained unclear. In this study, we observed that *KRAS* mutation frequency in pancreatic tumors was consistent with ours and other previous European studies that were based on 70–100 tumors and reported a mutation frequency of 72–83% [14], [16], [17]. Earlier, a Korean study on paraffin embedded 136 tumors reported a mutation frequency of 52% [18]. *KRAS* mutations in pancreatic cancer are believed to be the early events in neoplastic transformation. The hypothesis is supported by mice models based on conditional endogenous expression of the mutant *KRAS*. Those mouse models were developed with an assumption that *KRAS* mutation is an essential and early somatic genetic alteration in PDAC progression. Similar observations were reported for *KRAS* mutations in human acinar-ductal metaplasia (ADM) lesions of pancreas [19]. The ADM were purported to be the originating lesions for PDAC in mouse models [20]. Analysis of human ADM lesions showed that *KRAS* mutations existed only in the lesions associated with PanIN; the isolated ADM lesions were devoid of any *KRAS* mutation, with possible involvement of two distinct mechanisms, with and without *KRAS* mutations [19]. Those observations indicate that the presence of a *KRAS* mutation may not be essential for human PDAC progression and other low frequency gene mutations could trigger alternate pathways. The pancreatic genome sequencing of 20,661 genes from 24 tumors identified other low frequency gene mutations [21]. A recent study reported occurrence of 3–4 driver mutations in the *KRAS, CDKN2A, TP53* and *SMAD4* genes in about 30% of pancreatic tumors [15].

The codons 12, 13 and 61 of *KRAS* gene are part of the conserved ‘G-domain’ (residues 1–165) required for signal transduction. Tumor malignancy depends not on the presence of a *KRAS* mutation but on the molecular configuration and constituent mutation type [22]. Our data in this study showed that presence of any *KRAS* mutation in pancreatic tumors was associated with reduced survival time. Further analysis showed that the association was significant only for G12D sub-type of *KRAS* mutation. Lack of statistical power owing to low frequency of other mutation types likely precluded observation of the effect on survival. While our data being concordant with the paradigm of distribution of *KRAS* mutations, we clearly showed that patients, in particular, those harboring G12D mutation in tumors were at a 2-fold increased risk of death compared to those without any *KRAS* mutation. In an experimental study, the human cell lines with *KRAS* mutations were classified into *KRAS* dependent and independent. The ‘classical’ PDAC categorized as *KRAS* dependent were shown to be potentially amenable to the directed therapy [2], [23]. The importance such studies is underlined by the fact that mutational status in metastatic colorectal cancer is already an approved clinical tool for treatment with epidermal growth factor receptor monoclonal antibodies, cetuximab or panitumumab; as mutant *KRAS* has been established as a predictor of resistance to the treatment [24], [25].

The association between pancreatic cancer patient survival and specific sub-types of *KRAS* mutations could also be due to varying abilities to alter the RAS protein. *KRAS* mutations at codon 12, in general, have been shown to increase resistance to apoptosis and activate AKT/protein kinase B pathway [22]. In transgenic mice, the pancreas-specific and reversible expression of inducible *KRAS* G12D mutant was shown not only to initiate neoplastic lesions but was also involved in tumor maintenance [26]. In genetically engineered mice G12D mutant *KRAS* is reported to promote widespread colonic epithelia hyperplasia and neoplasia [27]. To best of our knowledge, this is the first report showing a clear association between *KRAS* mutation subtypes and survival. Our previous report on paraffin embedded tumors did not show any association between the presence of *KRAS* mutations and patient survival; however, there was difference in survival between the patients with different mutation types [14]. Lack of *KRAS* mutational status as predictive of survival was also reported in an earlier trial study of Gemctabine and Erlotinib therapy in patients with advanced pancreatic cancer [28]. *KRAS* mutations in the surgically negative resected margins have also been shown to be associated with clinical cancer recurrence, aggressive tumor biology and poor survival [29]. Similarly, detection of *KRAS* mutations in retroperitoneal margins, in the patients with complete pancreatectomy also showed poor prognosis [29].

The other gene that has been consistently reported to carry high frequency of somatic mutation in pancreatic cancers is *CDKN2A* [30]. The deletion/mutation frequency of *CDKN2A* in the present study was in agreement with that reported in the COSMIC database [31]. A mouse model with a conditional knock-in and knock-out of *Kras^G12D^* and *Ink4a/Arf* showed enhanced progression of pre-malignant lesions to PDAC [32], [33]. In this study we found that the subset of patients with concomitant *KRAS* and *CDKN2A* aberrations were at 2.5-fold higher risk of death than patients without any alterations in the two genes. In a previous study it was shown that 1–2 mutations in pancreatic tumors showed a median survival of 23 months compared to 13 months in our present study [15]. The difference in median survival can be, possibly, attributed to the fact that 149 out of 159 patients in our study had stage III and IV tumors. Mice models have shown that survival times were dependent on genetic aberrations accompanying a *KRAS* mutation [34], [35]. Similar results were reported in a study on *KRAS* mutations together with loss of heterozygosity on different chromosomal positions [29].

In conclusion, our results show that mutations in *KRAS* are frequent but not universal in pancreatic tumors and the presence of *KRAS* mutations in general, and G12D transformation in particular, were indicative of association with poor survival. Our results also showed that concomitant occurrence of *KRAS* mutations and aberrations in *CDKN2A* resulted in a sub-group of patients with lowest survival. Our data from this study is suggestive for a case for the prognostic classification of pancreatic cancer patients based on mutational status of *KRAS* and *CDKN2A.* However, the results need independent confirmation in additional studies with definite statistical confidence.

## Materials and Methods

### Ethics Statement

For all samples analyzed, written informed consent was obtained from the patients. The study was approved by the local ethics committee of the University of Heidelberg.

### Study population

Tumor tissues were collected from pancreatic cancer patients during surgery between January 2002 and September 2009, snap-frozen in liquid nitrogen directly after resection and subsequently stored at −80 °C. A total of 171 tumor tissues, that contained at least 10% tumor by H&E staining were analyzed in the present study. The clinical and histopathological characteristics of the patients are given in Table 1. The cell lines A549, SW1116, SW620, HS766T, MiaPaCa and LoVo were commercially obtained from American Type Culture collection (ATCC) [36], [37].

### Histopathological assessment of cellular composition of tissue biopsies

Three different tissue sections were selected randomly for hematoxylin and eosin (H&E) staining and histological validation. Slides were scanned with the ScanScope GL System (Aperio Technologies, Vista, CA, USA) and visualized using the ImageScope Software. For each tissue sample, three pathologists evaluated independently the histology and percentage of normal, tumor and stroma cells (Figure S1). Only samples with more than 10% tumor cells were pursued further.

### Genomic DNA extraction

Frozen pancreatic tissue samples were individually cut into 20 µm thick slices with a cryotome Leica CM 1850 UV at −34 °C. The tissue slices were covered with liquid nitrogen and gently ground by three turns with a micropestle made of polypropylene (Eppendorf, Hamburg, Germany) that fitted into 2 ml Eppendorf tubes. DNA from tissue slices and from cell lines was extracted using the AllPrep Isolation Kit (Qiagen, Hilden, Germany). DNA from cell lines with known *KRAS* mutations in codon 12, 13 and 61 were used as controls that included, A549 cell line with G12S (GGT>AGT) mutation; MiaPaCa, G12C (GGT>TGT); SW 1116, G12A (GGT>GCT); SW 620 G12V (GGT>GTT); LS-174, G12D (GGT>GAT); LoVo, G13D (GGC>GAC); HS 766T, Q61H (CAA>CAC). DNA samples from healthy controls were included as negative control.

### PCR, single strand conformation polymorphism (SSCP) and sequencing

PCR was carried out in 10 µl volume reactions using 10 ng of genomic DNA, 2 mM MgCl_2_, 0.11 mM each dNTP, 1 µCi [α-^32^P] dCTP, 0.2 µM each gene specific primer (Table S1), and 0.3 U Genaxxon Hot-start polymerase. The reactions were carried out in 35 cycles. Electrophoresis of the amplified fragments for SSCP was carried out on non-denaturing 0.5x MDE PAGE gels under at least 4 different conditions (Table S1). Each experiment was repeated twice and only when results were reproducible, shifted bands due to mutations were subjected to sequencing. The sequencing was carried out using a BigDye Terminator Cycle sequencing kit (Applied Biosystems). Amplified PCR product was treated with ExoSapIT (Amersham Biosciences, Uppsala, Sweden) and sequencing reactions were carried out in 10 µl reaction volumes using forward and reverse primers separately. The reaction products were analyzed on an ABI prism 3100 Genetic analyzer (Applied Biosystems).

### Multiplex ligation-based probe amplification (MLPA)

MLPA was used to detect homozygous deletions at the *CDKN2A* locus using the MLPA ME024A kit (MRC-Holland, Amsterdam, The Netherlands) which contained 30 probes mapping chromosome 9p21 and 9p22, 13 reference probes and 9 internal controls. Reference probes were located in genomic regions with low frequency copy number changes. The hybridization and ligations were carried out as per instructions and fragment analysis was performed on an ABIPRISM 3130xl capillary sequencer. The data were visualized using peak scanner v1.0 software and the exported data was analyzed with Coffalyser software v8 (MRC-Holland, Amsterdam, the Netherlands). Calculation of signal ratios was carried out as described by Mistry et al. [38]. Stringent criteria were adopted for data analysis using Coffalyser software and experiments were repeated twice for reproducibility.

### Statistical analyses

Of 171 tumors that were analyzed for mutations, 163 were malignant and 8 non-malignant tumors. Of the 163 patients with malignant tumors, survival data were available for 153 patients, of whom 150 patients had malignant tumors of pancreatic origin including ductal adenocarcinomas (n = 135) and rare carcinomas (n = 15). The rest (n = 3) were carcinoma of ampulla of Vater (Table 1). The Kaplan–Meier method was employed to determine the cumulative survival curves using time period (in months) between date of operation and the date of death. Differences between the groups were analyzed by the log-rank test. Univariate and multivariate Cox regression analyses were used to determine proportional hazard ratios. For multivariate analysis variables included were gender, age at surgery, TNM status, tumor differentiation grade and histological status of tumors. All statistical analyses were carried out by using SAS® version 9.2 (SAS Institute Inc., Cary, NC).

## Supporting Information

Figure S1 **Histomorphological examination of pancreatic tumor tissue sections with Hematoxylin and Eosin stains.** Representative photomicrographs of three sections with low, medium and high tumor contents are shown.
(TIF)

Figure S2 **Representative SSCP of** ***KRAS*** **codon 12 and codon 61 in pancreatic tumors.** (A) The lanes 1–4 contain amplified fragments of exon 2 (codon 12) and lanes 5–6 contain amplified fragments of exon 3 (codon 61) from tumor DNA samples. The shifted bands seen in lane 1 contain GGT>GAT (G12D) mutation, lane 2 contains GGT>CGT (G12R), lane 3 contains GGT>GTT (G12V) mutation and lane 4 contains tumor DNA without mutation in exon 2. The shifted bands in lane 5 contain CAA>CAC (Q61H) mutation and lane 6 contains tumor DNA without mutation in exon 3. **(B)** Sequence analysis of a part of exon 2 of *KRAS* gene (coding strand) with GGT>GAT (G12D) mutation. **(C)** A part of exon 2 sequence showing GGT>CGT (G12R) mutation. **(D)** A part of exon 2 sequence showing GGT>GTT (G12V) mutation. **(E)** A part of the exon 2 showing wild type sequence at codon 12 and codon of *KRAS*. **(F)** A part of exon 3 sequence showing CAA>CAC (Q61H) mutation. **(G)** A part of the exon 3 showing the wild type sequence at codon 61 of *KRAS*.
(TIF)

Figure S3 **Kaplan-Meier survival curves showing difference in overall survival in exocrine cancer patients with and without mutations. (**A) Median survival of patients with *KRAS* mutations was 17 months against 30 months for patients without mutations in the gene. (B) Median survival of patients with *KRAS* codon 12 GGT>GAT (G12D) mutations was 16 months against 30 months for patients without any mutation in *KRAS*. (C) Median survival of patients with concomitant alterations in *KRAS* and *CDKN2A* genes was 13 months against 30 months for patients without any alterations in both *KRAS* and *CDKN2A*.
(TIF)

Table S1 Primer sequences and SSCP conditions for detection of mutations in the KRAS and CDKN2A genes.
(DOC)

Table S2 Mutation frequency by clinic pathology and effect on survival of pancreatic cancer patients.
(DOC)

Table S3 Clinico-pathological details and tumor mutational status of all pancreatic cancer patients.
(DOC)
