# Supplementary figures and images for: Somatic Mutations in Exocrine Pancreatic Tumors: Association with Patient Survival
Source: PLoS One. 2013 Apr 2;8(4):e60870. doi: 10.1371/journal.pone.0060870 (PMC3614935; doi:10.1371/journal.pone.0060870)

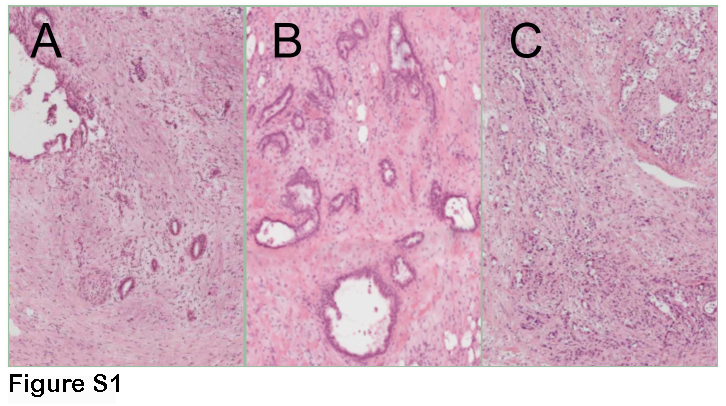

Supplement: Figure S1 — Histomorphological examination of pancreatic tumor tissue sections with Hematoxylin and Eosin stains. Representative photomicrographs of three sections with low, medium and high tumor contents are shown. (TIF) [file pone.0060870.s001.tif]

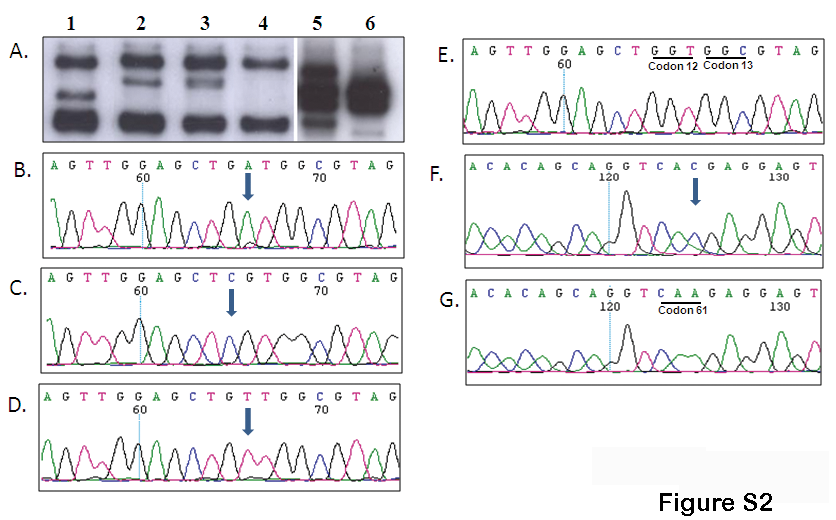

Supplement: Figure S2 — Representative SSCP of KRAS codon 12 and codon 61 in pancreatic tumors. (A) The lanes 1–4 contain amplified fragments of exon 2 (codon 12) and lanes 5–6 contain amplified fragments of exon 3 (codon 61) from tumor DNA samples. The shifted bands seen in lane 1 contain GGT>GAT (G12D) mutation, lane 2 contains GGT>CGT (G12R), lane 3 contains GGT>GTT (G12V) mutation and lane 4 contains tumor DNA without mutation in exon 2. The shifted bands in lane 5 contain CAA>CAC (Q61H) mutation and lane 6 contains tumor DNA without mutation in exon 3. (B) Sequence analysis of a part of exon 2 of KRAS gene (coding strand) with GGT>GAT (G12D) mutation. (C) A part of exon 2 sequence showing GGT>CGT (G12R) mutation. (D) A part of exon 2 sequence showing GGT>GTT (G12V) mutation. (E) A part of the exon 2 showing wild type sequence at codon 12 and codon of KRAS. (F) A part of exon 3 sequence showing CAA>CAC (Q61H) mutation. (G) A part of the exon 3 showing the wild type sequence at codon 61 of KRAS. (TIF) [file pone.0060870.s002.tif]

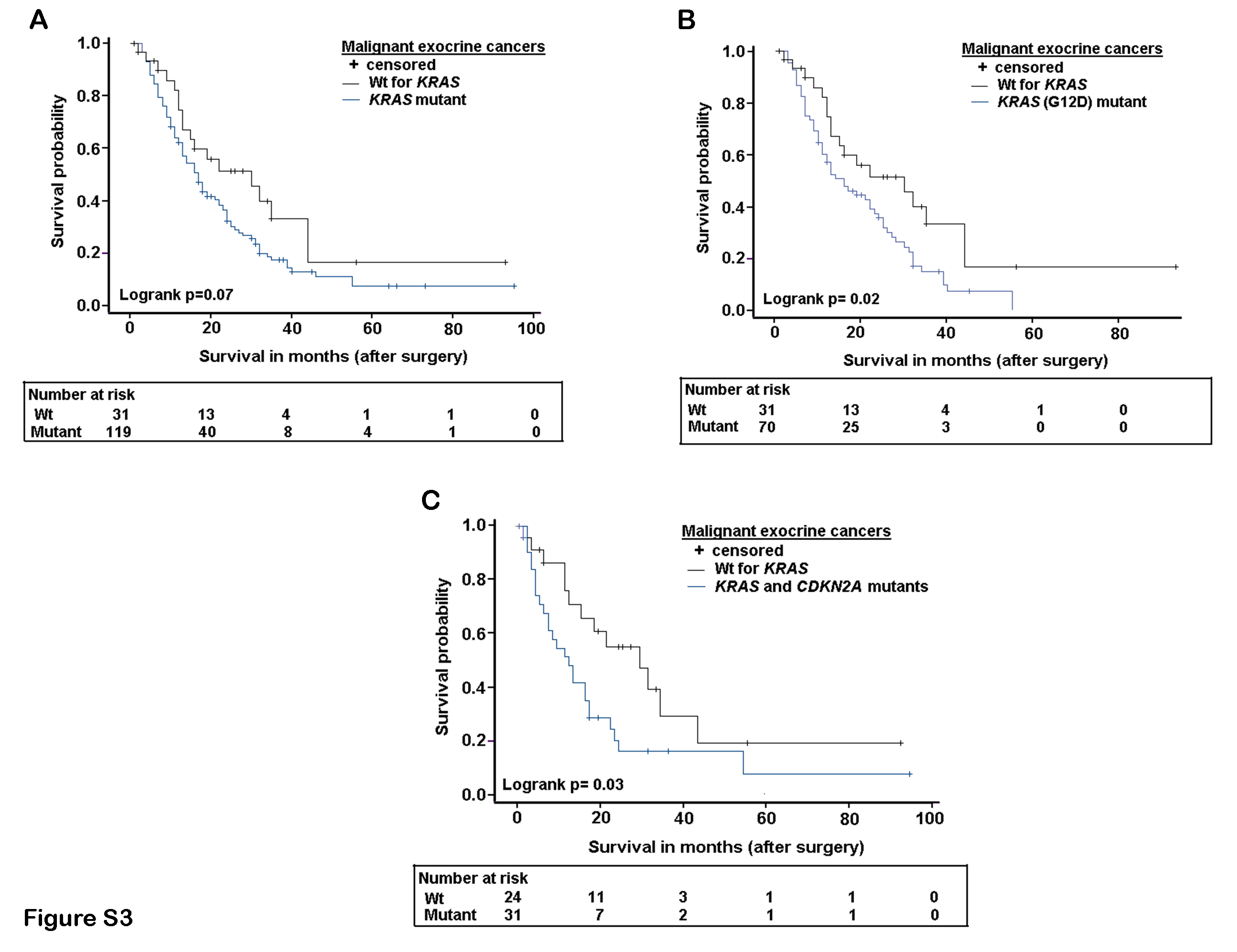

Supplement: Figure S3 — Kaplan-Meier survival curves showing difference in overall survival in exocrine cancer patients with and without mutations. (A) Median survival of patients with KRAS mutations was 17 months against 30 months for patients without mutations in the gene. (B) Median survival of patients with KRAS codon 12 GGT>GAT (G12D) mutations was 16 months against 30 months for patients without any mutation in KRAS. (C) Median survival of patients with concomitant alterations in KRAS and CDKN2A genes was 13 months against 30 months for patients without any alterations in both KRAS and CDKN2A. (TIF) [file pone.0060870.s003.tif]
