# Supplementary material for: Somatic Mutations in Exocrine Pancreatic Tumors: Association with Patient Survival
Source: PLoS One. 2013 Apr 2;8(4):e60870. doi: 10.1371/journal.pone.0060870 (PMC3614935; doi:10.1371/journal.pone.0060870)
Supplement: Table S1 — Primer sequences and SSCP conditions for detection of mutations in the KRAS and CDKN2A genes. (DOC) [file pone.0060870.s004.doc]

**Table S1.** **Primer sequences and SSCP conditions for detection of mutations in the *KRAS* and *CDKN2A* genes**

|  | ***KRAS* (exon 2: codon 11, 12 and 13)** | ***KRAS* (exon 3: codon 61)** |  |
| --- | --- | --- | --- |
| Fragment length | 161bp | 130bp |  |
| Forward primer | ATTATTTTTATTATAAGGCCTGCTG | CCAGACTGTGTTTCTCCCTTC |  |
| Reverse primer | TGCATATTAAAACAAGATTTAC | AAAGAAAGCCCTCCCCAGT |  |
| PCR annealing | 54°C | 60°C |  |
| SSCP condition | GAT, GTT, CGT (RT without glycerol) GAT (16°C) | 4°C (without glycerol) |  |
|  |  |  |  |
|  | ***CDKN2A* (p16) Exon 1α** | ***CDKN2A* (p16) Exon 2 (5’end)** | ***CDKN2A* (p16) Exon 2 (3’end)** |
| Fragment length | 227bp | 204bp | 260bp |
| Forward primer | GGAGAGCAGGCAGCGGGC | CTACACAAGCTTCCTTTCCGTCAT | ACCCCGCCACTCTCACC |
| Reverse primer | AGAGTCGCCCGCCATCCCC | CCACCAGCGTGTCCAGGAA | ATTCTCAGATCATCAGTCCTCAC |
| PCR annealing | 68°C | 59°C | 62 °C |
| SSCP condition | 4°C (without glycerol) | RT (without glycerol) | RT (without glycerol) |
