# Supplementary material for: Somatic Mutations in Exocrine Pancreatic Tumors: Association with Patient Survival
Source: PLoS One. 2013 Apr 2;8(4):e60870. doi: 10.1371/journal.pone.0060870 (PMC3614935; doi:10.1371/journal.pone.0060870)
Supplement: Table S2 — Mutation frequency by clinic pathology and effect on survival of pancreatic cancer patients. (DOC) [file pone.0060870.s005.doc]

**Table S2. Mutation frequency by clinic pathology and effect on survival of pancreatic cancer patients**

|  |  | ***KRAS*** | | | | **CDKN2A** |
| --- | --- | --- | --- | --- | --- | --- |
|  |  | **Wt** | **Mutation (%**) | **P-value^*^** | **HR (95% CI)^*^** | **Mutation (%)** |
| **All categories** |  | 37 | 134 (78) | 0.07 | 1.58 (0.96-2.62) | 43 (25) |
| **Gender** | Male | 100 | 79 (79) | 0.18 | 1.62 (0.80-3.27) | 21 (21) |
|  | Female | 71 | 55 (77) | 0.13 | 1.76 (0.84-3.72) | 22 (31) |
| **Age at surgery (years)** | Median**=** 65 (56-70) (Mean= 63 ± 11.31) | 171 | 134 (78) |  |  | 43 (25) |
| **Histologic variants** |  |  |  |  |  |  |
| benign | Serous cystadenoma, SCA | 4 | 1 (25) |  | - | - |
| premalignant | Mucinous cystic neoplasm, MCN | 1 | - |  | - | 1 (100) |
|  | Intraductal papillary mucinous neoplasm, IPMN (low grade) | 3 | 3 (100) |  | - | - |
| malignant | **Ductal adenocarcinomas** |  |  | 0.16 |  |  |
|  | PDAC | 134 | 111 (83) |  |  | 32 (24) |
|  | Adenosquamous carcinoma | 5 | 3 (60) |  |  | 3 (100) |
|  | Anaplastic undifferentiated carcinoma | 4 | 3 (75) |  |  | 3 (100) |
|  | **Carcinomas: rare cases** |  |  | 0.73 | 1.54 (0.14-17.15) |  |
|  | Acinar cell carcinoma | 2 | - |  |  | 1 (50) |
|  | Microcystic tubulopapillary adenocarcinoma | 2 | 1 (50) |  |  | 2 (100) |
|  | Intraductal papillary mucinous neoplasm, IPMN (invasive carcinoma) | 9 | 7 (78) |  |  | 1 (11) |
|  | SPN/Frantz’s tumor | 2 | - |  |  | - |
|  | Cystadenocarcinoma | 1 | 1 (100) |  |  | - |
| ampullary region | **Carcinoma of ampulla Vateri** | 4 | 4 (100) |  | - | - |
| **Tumor location** | Pancreatic head | 111 | 87 (78) | 0.51 | 1.20 (0.70-2.07) | 26 (23) |
|  | Pancreatic body | 19 | 15 (79) | 0.52 | 1.67 (0.35-8.00) | 4 (21) |
|  | Pancreatic tail | 20 | 16 (80) |  | - | 7 (35) |
|  | Overlapping sites | 13 | 9 (69) |  | - | 5 (38) |
|  | Ampulla Vateri | 4 | 4 (100) |  | - | 1 |
| **TNM status** | Tis (T0) | 3 | 1 (50) |  | - | 1 (33) |
|  | T1 | 3 | 3 (100) |  | - | 1 (33) |
|  | T2 | 2 | 1 (100) |  | - | - |
|  | T3 | 130 | 107 (82) | 0.03 | 1.94 (1.05-3.56) | 34 (26) |
|  | T4 | 19 | 17 (89) | 0.20 | 0.35 (0.07-1.76) | 4 (21) |
|  | no status | 14 | 5 |  |  | 3 |
|  | N0 | 31 | 22 (71) | 0.54 | 1.49 (0.42-5.23) | 10 (32) |
|  | N1 | 128 | 107 (84) | 0.12 | 1.54 (0.89-2.68) | 30 (23) |
|  | no status | 12 | 5 |  |  | 3 |
|  | M0 | 141 | 112 (79) | 0.12 | 1.54 (0.89-2.68) | 35 (25) |
|  | M1 | 18 | 17 (94) |  | - | 5 (28) |
|  | no status | 12 | 5 |  |  | 3 |
| **Grade** | G1 | 7 | 3 (43) |  | - | 3 (43) |
|  | G2 | 88 | 73 (83) | 0.46 | 1.35 (0.62-2.96) | 19 (22) |
|  | G3 | 57 | 48 (83) | 0.06 | 2.50 (0.97-6.41) | 14 (27) |
|  | no status | 15 | 8 |  |  | 4 |
|  | Anaplastic type | 4 | 3 (50) |  | - | 3 (50) |

* Univariate hazard ratio and corresponding P-value for the effect of KRAS mutations on patient survival
