# Supplementary material for: Somatic Mutations in Exocrine Pancreatic Tumors: Association with Patient Survival
Source: PLoS One. 2013 Apr 2;8(4):e60870. doi: 10.1371/journal.pone.0060870 (PMC3614935; doi:10.1371/journal.pone.0060870)
Supplement: Table S3 — Clinico-pathological details and tumor mutational status of all pancreatic cancer patients. (DOC) [file pone.0060870.s006.doc]

**Table S3.** **Clinico-pathological details and tumor mutational status of all pancreatic cancer patients**

| ***** Patient ID | Gender | Age (years) | Survival (months) | Census status | Histology | Localization | Grade | T | N | M | *KRAS* | *CDKN2A* |
| --- | --- | --- | --- | --- | --- | --- | --- | --- | --- | --- | --- | --- |
| 107 | F | 51 | 34 | Alive | Acinar cell carcinoma | head region | 1 | 3 | 0 | 0 | wild type | wild type |
| 300 | M | 43 | 35 | Alive | Acinar cell carcinoma | tail region | 3 | 3 | 1 | 1 | wild type | deletion |
| 25 | M | 66 | 13 | Dead | Adenosquamous carcinoma | head region | 3 | 3 | 1 | 0 | wild type | wild type |
| 156 | M | 66 | 13 | Dead | Adenosquamous carcinoma | head region | 3 | 3 | 1 | 0 | wild type | deletion |
| 189 | F | 54 | 17 | Dead | Adenosquamous carcinoma | head region | 3 | 3 | 1 | 0 | GGT>GAT (G12D) | wild type |
| 204 | F | 71 | 3 | Dead | Adenosquamous carcinoma | head region | 2 | 3 | 0 | 0 | GGT>GAT (G12D) | deletion |
| 269 | M | 80 | 2 | Dead (other cause) | Adenosquamous carcinoma | body region | 3 | 3 | 0 | 0 | GGT>GAT (G12D) | deletion |
| 106 | M | 63 | 2 | Dead | Anaplastic undifferentiated carcinoma | head region | 4 | 3 | 1 | 0 | wild type | wild type |
| 238 | M | 65 | 3 | Dead | Anaplastic undifferentiated carcinoma | tail region | 4 | 3 | 0 | 0 | GGT>GAT (G12D) | deletion |
| 527 | M | 67 | . | no follow up | Anaplastic undifferentiated carcinoma | head region | 4 | 3 | 1 | 0 | GGT>CGT (G12R) | deletion |
| 997 | F | 64 | 4 | Dead | Anaplastic undifferentiated carcinoma | head region | 4 | 3 | 1 | 0 | GGT>GAT (G12D) | deletion |
| 126 | F | 56 | 14 | Dead | carcinoma of ampulla-Vateri | ampulla-Vateri | 2 | 4 | 0 | 0 | GGT>TGT (G12C) | wild type |
| 195 | M | 55 | 0 | Alive | carcinoma of ampulla-Vateri | ampulla-Vateri | 3 | 4 | 1 | 0 | GGT>GAT (G12D) | wild type |
| 513 | M | 63 | 25 | Alive | carcinoma of ampulla-Vateri | ampulla-Vateri | 2 | 4 | 1 | 0 | GGT>GAT (G12D) | wild type |
| 681 | M | 45 | . | no follow up | carcinoma of ampulla-Vateri | ampulla-Vateri | 2 | 4 | 1 | 0 | GGT>GAT (G12D) | wild type |
| 407 | F | 43 | 30 | Alive | Cystadenocarcinoma | tail region | 2 | 1 | 0 | 0 | GGT>GTT (G12V) | wild type |
| 370 | M | 68 | 21 | Alive | IPMN-benign (low grade) | head region | . | . | . | . | GGT>GAT (G12D) | wild type |
| 540 | F | 72 | 1 | Dead (other cause) | IPMN-benign (low grade) | head region | . | . | . | . | GGT>GAT (G12D) | wild type |
| 961 | M | 71 | . | non-apparent | IPMN-benign (low grade) | . | . | . | . | . | GGT>GAT (G12D) | wild type |
| 157 | M | 75 | 12 | Dead | IPMN-malignant (invasive carcinoma) | head region | 2 | 3 | 1 | 1 | GGT>GAT (G12D) | wild type |
| 186 | M | 72 | 39 | Dead | IPMN-malignant (invasive carcinoma) | body region | 2 | 3 | 0 | 0 | GGT>GAT (G12D) | wild type |
| 190 | M | 46 | 45 | Alive | IPMN-malignant (invasive carcinoma) | overlapping sites | 2 | 3 | 1 | 0 | GGT>GAT (G12D) | wild type |
| 383 | M | 67 | 4 | Dead (other cause) | IPMN-malignant (invasive carcinoma) | overlapping sites | 3 | 3 | 1 | 1 | GGT>GAT (G12D) | wild type |
| 657 | M | 75 | 95 | Alive | IPMN-malignant (invasive carcinoma) | . | . | 0 | 0 | 0 | GGT>TGT (G12C) | deletion |
| 675 | M | 65 | 2 | Alive | IPMN-malignant (invasive carcinoma) | head region | 2 | 0 | 0 | 0 | wild type | wild type |
| 679 | M | 50 | 20 | Alive | IPMN-malignant (invasive carcinoma) | head region | 2 | 0 | 0 | 0 | wild type | wild type |
| 950 | F | 67 | 12 | Alive | IPMN-malignant (invasive carcinoma) | tail region | 2 | 1 | 0 | 0 | GGT>GAT (G12D) | wild type |
| 996 | M | 67 | 6 | Dead | IPMN-malignant (invasive carcinoma) | head region | 2 | 3 | 1 | 0 | GGT>GAT (G12D) | wild type |
| 139 | M | 47 | 43 | Alive | MCN- benign | tail region | . | . | . | . | wild type | deletion |
| 172 | F | 53 | 15 | Dead | Microcystic tubulopapillary adenocarcinoma | head region | . | . | . | . | wild type | deletion |
| 217 | M | 60 | 32 | Alive | Microcystic tubulopapillary adenocarcinoma | head region | . | 3 | 0 | 0 | GGT>GAT (G12D) | deletion |
| 5 | F | 67 | 11 | Dead | PDAC | head region | 2 | 4 | 0 | 0 | wild type | deletion |
| 8 | F | 66 | 4 | Dead | PDAC | head region | 3 | 3 | 0 | 0 | CAA>CAC (Q61H) | deletion |
| 14 | F | 58 | 9 | Dead | PDAC | head region | 2 | 4 | 1 | 0 | GGT>GAT (G12D) | wild type |
| 15 | F | 41 | 24 | Dead | PDAC | head region | 2 | 3 | 1 | 0 | GGT>CGT (G12R) | wild type |
| 18 | M | 65 | 46 | Dead | PDAC | head region | 2 | 3 | 1 | 0 | GGT>CGT (G12R) | wild type |
| ****** 21 | M | 56 | 24 | Dead | PDAC | head region | 2 | 3 | 1 | 0 | GGT>CGT (G12R) | c. 78insGG |
| 22 | F | 60 | 44 | Dead | PDAC | head region | 3 | 3 | 1 | 0 | wild type | wild type |
| 23 | M | 55 | 18 | Dead | PDAC | head region | 2 | 3 | 1 | 0 | GGT>CGT (G12R) | wild type |
| 24 | M | 47 | 13 | Dead | PDAC | head region | 2 | 3 | 1 | 0 | GGT>GAT (G12D) | wild type |
| 28 | M | 59 | 24 | Dead | PDAC | head region | . | . | . | . | GGT>GTT (G12V) | wild type |
| 32 | F | 67 | 12 | Dead | PDAC | head region | 3 | 3 | 1 | 0 | wild type | wild type |
| ****** 35 | M | 57 | 39 | Dead | PDAC | head region | 2 | 3 | 1 | 0 | GGT>GAT (G12D) | wild type |
| 40 | M | 72 | 7 | Dead | PDAC | head region | 2 | 3 | 1 | 0 | GGT>CGT (G12R) | wild type |
| 41 | F | 70 | 12 | Dead | PDAC | head region | 3 | 3 | 1 | 1 | GGT>GAT (G12D) | deletion |
| ****** 43 | F | 77 | 66 | Alive | PDAC | head region | 2 | 3 | 1 | 0 | GGT>CGT (G12R) | wild type |
| 44 | M | 76 | 64 | Alive | PDAC | head region | 2 | 3 | 0 | 0 | GGT>CGT (G12R) | wild type |
| 45 | M | 66 | 13 | Dead | PDAC | head region | 1 | 3 | 1 | 0 | GGT>CGT (G12R) | deletion |
| 46 | M | 61 | 32 | Dead | PDAC | head region | 2 | 3 | 1 | 0 | GGT>GAT (G12D) | wild type |
| 48 | F | 72 | 13 | Dead | PDAC | head region | 2 | 3 | 0 | 0 | GGT>GTT (G12V) | wild type |
| 61 | F | 52 | 44 | Dead | PDAC | head region | 1 | 3 | 1 | 0 | wild type | deletion |
| 66 | M | 76 | 16 | Dead | PDAC | head region | 3 | 3 | 1 | 1 | GGT>GAT (G12D) | wild type |
| 71 | F | 66 | 8 | Dead | PDAC | tail region | 3 | 3 | 1 | 0 | GGT>GTT (G12V) | wild type |
| 72 | M | 61 | 30 | Dead | PDAC | head region | 2 | 3 | 1 | 0 | wild type | wild type |
| 77 | F | 56 | 4 | Dead | PDAC | head region | 2 | 4 | 1 | 0 | wild type | wild type |
| 78 | M | 63 | 93 | Alive | PDAC | overlapping sites | 2 | 3 | 1 | 0 | wild type | wild type |
| 80 | F | 70 | 7 | Dead | PDAC | head region | 2 | 3 | 1 | 0 | GGT>GAT (G12D) | deletion |
| 94 | M | 81 | 40 | Dead | PDAC | head region | 3 | 3 | 1 | 0 | GGT>GAT (G12D) | wild type |
| 95 | F | 66 | 4 | Dead | PDAC | head region | 3 | 3 | 0 | 0 | GGT> GCT (G12A ) | wild type |
| 98 | F | 66 | 16 | Dead (other cause) | PDAC | body region | 3 | 3 | 1 | 1 | GGT>GTT (G12V) | wild type |
| 100 | M | 60 | 5 | Dead | PDAC | head region | 3 | 3 | 1 | 0 | GGT>GTT (G12V) | wild type |
| 101 | M | 66 | 27 | Dead | PDAC | body region | 2 | 3 | 1 | 1 | GGT>GAT (G12D) | wild type |
| 104 | F | 78 | 10 | Dead | PDAC | overlapping sites | 2 | 3 | 1 | 0 | GGT>GAT (G12D) | CGA>TGA (R80*) |
| 105 | M | 70 | 14 | Dead | PDAC | head region | 3 | 3 | 0 | 0 | GGT>GTT (G12V) | deletion |
| 109 | M | 52 | 16 | Dead | PDAC | head region | 1 | 3 | 1 | 0 | wild type | wild type |
| 120 | M | 58 | 35 | Dead | PDAC | head region | 2 | 2 | 1 | 0 | wild type | wild type |
| 143 | M | 56 | 7 | Dead | PDAC | head region | 3 | 3 | 1 | 0 | GGT>GAT (G12D) | wild type |
| 145 | M | 60 | 26 | Dead | PDAC | head region | 3 | 2 | 0 | 0 | GGT>GAT (G12D) | wild type |
| 148 | M | 55 | 12 | Dead | PDAC | head region | 3 | . | . | . | wild type | wild type |
| 151 | F | 59 | 35 | Dead | PDAC | head region | 2 | 3 | 1 | 1 | CAA>CAC (Q61H) | wild type |
| 154 | M | 67 | 8 | Dead | PDAC | head region | 2 | 4 | 1 | 0 | GGT>GAT (G12D) | deletion |
| 160 | M | 61 | 11 | Dead | PDAC | head region | 2 | 4 | 1 | 0 | GGT>GAT (G12D) | wild type |
| 163 | M | 56 | 34 | Dead | PDAC | head region | 2 | 3 | 1 | 0 | GGT>GAT (G12D) | wild type |
| 164 | M | 43 | 28 | Dead | PDAC | head region | 2 | 3 | 1 | 0 | GGT>GAT (G12D) | wild type |
| 165 | M | 50 | 32 | Dead | PDAC | head region | 3 | 3 | 1 | 0 | GGT>GAT (G12D) | wild type |
| 166 | M | 75 | 13 | Dead | PDAC | head region | 2 | 4 | 1 | 1 | GGT>CGT (G12R) | wild type |
| 167 | F | 40 | 25 | Dead | PDAC | body region | 2 | 3 | 1 | 0 | GGT>GAT (G12D) | wild type |
| ****** 180 | M | 62 | 18 | Dead | PDAC | head region | 2 | 3 | 1 | 0 | GGT>GTT (G12V) | deletion |
| 182 | F | 59 | 18 | Dead | PDAC | tail region | 2 | 3 | 1 | 0 | GGT>CGT (G12R) | deletion |
| 183 | M | 68 | . | no follow up | PDAC | body region | 2 | 3 | 1 | 0 | wild type | wild type |
| 184 | F | 44 | 3 | Dead | PDAC | body region | 2 | 3 | 1 | 0 | GGT>GTT (G12V) | wild type |
| 185 | M | 67 | 16 | Dead | PDAC | body region | 2 | 3 | 0 | 0 | GGT>GTT (G12V) | wild type |
| 196 | M | 68 | 55 | Dead | PDAC | head region | 2 | 3 | 1 | 0 | GGT>GAT (G12D) | deletion |
| 197 | F | 58 | 23 | Dead | PDAC | tail region | 2 | 3 | 1 | 0 | GGT>GAT (G12D) | wild type |
| 208 | M | 70 | 11 | Dead | PDAC | head region | 3 | 3 | 1 | 0 | GGT>GAT (G12D) | wild type |
| 216 | F | 54 | 5 | Dead | PDAC | body region | 1 | 1 | 0 | 0 | GGT>GAT (G12D) | deletion |
| 219 | F | 73 | 5 | Dead | PDAC | head region | 2 | 3 | 1 | 0 | GGT>GAT (G12D) | wild type |
| 229 | F | 88 | 6 | Dead | PDAC | tail region | 3 | 3 | 1 | 0 | CAA>CAC (Q61H) | wild type |
| 234 | M | 56 | 3 | Dead | PDAC | tail region | 3 | 3 | 1 | 0 | GGT>GAT (G12D) | deletion |
| 235 | M | 51 | 30 | Dead | PDAC | head region | 3 | 3 | 0 | 0 | GGT>GAT (G12D) | wild type |
| 254 | M | 55 | 10 | Dead | PDAC | head region | 2 | 4 | 1 | 0 | GGT>GAT (G12D) | wild type |
| 266 | M | 66 | 6 | Dead | PDAC | overlapping sites | 3 | 3 | 1 | 0 | GGT>GAT (G12D) | wild type |
| 277 | M | 53 | 19 | Dead | PDAC | tail region | 2 | 4 | 0 | 0 | GGT>GAT (G12D) | wild type |
| 288 | F | 55 | . | no follow up | PDAC | tail region | 3 | 3 | 1 | 1 | GGT>GAT (G12D) | deletion |
| 301 | M | 76 | 16 | Dead | PDAC | head region | 3 | 3 | 1 | 1 | GGT>GAT (G12D) | wild type |
| 302 | F | 66 | 8 | Dead | PDAC | tail region | 3 | 3 | 1 | 0 | GGT>GTT (G12V) | wild type |
| 322 | F | 64 | 9 | Dead | PDAC | overlapping sites | 2 | 3 | 1 | 1 | GGT>GAT (G12D) | deletion |
| 325 | M | 69 | 31 | Dead | PDAC | head region | 2 | 3 | 1 | 0 | GGT>CGT (G12R) | wild type |
| 329 | M | 70 | 31 | Alive | PDAC | head region | 2 | 4 | 1 | 0 | GGT>CGT (G12R) | wild type |
| 340 | M | 68 | 7 | Dead | PDAC | head region | 3 | 3 | 1 | 0 | GGT>GAT (G12D) | wild type |
| 359 | F | 50 | 31 | Dead | PDAC | head region | 3 | 3 | 1 | 0 | GGT>GAT (G12D) | wild type |
| 360 | M | 64 | 17 | Dead | PDAC | body region | 2 | 3 | 1 | 0 | GGT>GTC (G12V) | deletion |
| 382 | M | 52 | 16 | Dead | PDAC | head region | 2 | 3 | 1 | 0 | GGT>GTT (G12V) | wild type |
| 384 | F | 69 | 37 | Alive | PDAC | head region | 3 | 3 | 1 | 0 | GGT>GTT (G12V) | CAC>TAC (H83Y) |
| 402 | M | 59 | 40 | Alive | PDAC | head region | 3 | 3 | 1 | 0 | GGT>GTT (G12V) | wild type |
| 404 | M | 48 | . | no follow up | PDAC | head region | 1 | 3 | 1 | 0 | GGT>GTT (G12V) | wild type |
| 405 | F | 73 | 9 | Dead | PDAC | body region | 2 | 4 | 1 | 0 | GGT>CGT (G12R) | wild type |
| 408 | F | 78 | 11 | Dead | PDAC | head region | 2 | 3 | 1 | 0 | GGT>GTT (G12V) | wild type |
| 413 | F | 74 | 9 | Dead | PDAC | head region | 3 | . | . | . | wild type | deletion |
| 414 | F | 76 | 8 | Dead | PDAC | head region | 2 | 3 | 1 | 0 | GGT>GTT (G12V) | deletion |
| 416 | F | 74 | 24 | Alive | PDAC | head region | 2 | 3 | 0 | 0 | GGT>GAT (G12D) | wild type |
| 417 | F | 66 | 19 | Dead | PDAC | head region | 3 | 3 | 1 | 0 | GGT>GTT (G12V) | wild type |
| 421 | M | 78 | 7 | Dead | PDAC | head region | 2 | 3 | 1 | 0 | wild type | wild type |
| 511 | M | 64 | 19 | Alive | PDAC | head region | 2 | 4 | 1 | 0 | GGT>GAT (G12D) | wild type |
| 514 | F | 57 | 20 | Alive | PDAC | head region | 2 | 3 | 1 | 0 | GGT>GTT (G12V) | wild type |
| 517 | F | 66 | 17 | Dead | PDAC | tail region | 2 | 3 | 1 | 1 | GGT>CGT (G12R) | wild type |
| 519 | M | 86 | 11 | Dead | PDAC | head region | 3 | 3 | 1 | 0 | GGT>GTT (G12V) | wild type |
| 520 | M | 68 | 14 | Dead | PDAC | tail region | 3 | 3 | 1 | 0 | GGT>GAT (G12D) | wild type |
| 534 | M | 57 | 1 | Alive | PDAC | head region | 3 | 3 | 1 | 0 | GGT>GAT (G12D) | wild type |
| 537 | F | 69 | 17 | Dead | PDAC | head region | 2 | 4 | 1 | 0 | GGT>GTT (G12V) | TAC>TAA (Y129*) |
| 539 | M | 62 | 0 | Dead (other cause) | PDAC | body region | 3 | 4 | 1 | 0 | GGT>GAT (G12D) | wild type |
| 545 | M | 47 | 10 | Dead | PDAC | head region | 2 | 3 | 1 | 0 | GGT>CGT (G12R) | wild type |
| ******* 547 | F | 59 | 38 | Alive | PDAC | body region | 3 | 3 | 1 | 0 | GGT>GAT (G12D) | wild type |
| 548 | M | 70 | 5 | Dead | PDAC | body region | 3 | 3 | 1 | 1 | GGT>GAT (G12D) | CGA>TGA (R80*) |
| 550 | M | 77 | 6 | Dead | PDAC | head region | 3 | 3 | 1 | 0 | GGT>GAT (G12D) | CTG>CAG (L130Q) |
| 553 | M | 73 | 28 | Alive | PDAC | head region | 2 | 3 | 1 | 0 | wild type | wild type |
| 562 | M | 68 | 14 | Dead | PDAC | overlapping sites | 2 | 3 | 1 | 0 | GGT>CGT (G12R) | deletion |
| 660 | M | 54 | 17 | Alive | PDAC | body region | 2 | 3 | 1 | 1 | GGT>TGT (G12C) | wild type |
| 665 | M | 62 | 9 | Dead | PDAC | head region | 3 | 3 | 1 | 1 | GGT>CGT (G12R) | wild type |
| 668 | F | 67 | 18 | Alive | PDAC | tail region | 3 | 3 | 1 | 0 | GGT>CGT (G12R) | wild type |
| 669 | M | 77 | 12 | Alive | PDAC | head region | 3 | 3 | 1 | 0 | GGT>GAT (G12D) | wild type |
| 673 | F | 72 | . | no follow up | PDAC | overlapping sites | 2 | 3 | 1 | 0 | wild type | CAC>TAC (H83Y) |
| 677 | F | 79 | 20 | Alive | PDAC | head region | 3 | 3 | 1 | 0 | GGT>GAT (G12D) | wild type |
| 678 | M | 64 | 4 | Dead | PDAC | head region | 3 | 3 | 1 | 1 | GGT>GAT (G12D) | wild type |
| 680 | M | 68 | 10 | Alive | PDAC | overlapping sites | 3 | 3 | 1 | 0 | GGT>GAT (G12D) | wild type |
| 683 | M | 79 | 7 | Dead | PDAC | overlapping sites | 2 | 3 | 1 | 0 | GGT>GAT (G12D) | wild type |
| 687 | M | 63 | 13 | Dead | PDAC | head region | 2 | 3 | 1 | 0 | GGT>GAT (G12D) | wild type |
| 695 | M | 59 | 20 | Alive | PDAC | overlapping sites | 2 | 3 | 1 | 0 | GGT>GTT (G12V) | deletion |
| 821 | F | 77 | 21 | Dead | PDAC | head region | 3 | 3 | 1 | 0 | GGT>GAT (G12D) | wild type |
| 822 | M | 53 | 32 | Dead | PDAC | head region | 2 | 3 | 1 | 0 | GGT>GAT (G12D) | wild type |
| ****** 824 | F | 59 | 7 | Dead | PDAC | head region | 2 | 3 | 1 | 0 | GGT>GAT (G12D) | wild type |
| 832 | M | 47 | 13 | Dead | PDAC | head region | 2 | 3 | 1 | 0 | GGT>GAT (G12D) | wild type |
| ****** 838 | M | 67 | 22 | Dead | PDAC | body region | 2 | 3 | 1 | 0 | wild type | wild type |
| 841 | M | 77 | 56 | Alive | PDAC | head region | 3 | 3 | 1 | 0 | wild type | wild type |
| ****** 843 | M | 59 | 5 | Dead | PDAC | head region | 2 | 4 | 1 | 0 | GGT>CGT (G12R) | wild type |
| 844 | F | 73 | 32 | Dead | PDAC | body region | 1 | 3 | 0 | 0 | wild type | wild type |
| 847 | F | 61 | 25 | Dead | PDAC | head region | 2 | 4 | 1 | 0 | GGT>GAT (G12D) | deletion |
| 848 | F | 52 | 19 | Dead | PDAC | head region | 2 | 3 | 0 | 0 | wild type | wild type |
| 849 | M | 66 | 73 | Alive | PDAC | head region | 2 | 3 | 1 | 0 | GGT>CGT (G12R) | wild type |
| 850 | M | 55 | 18 | Dead | PDAC | head region | 2 | 3 | 1 | 0 | GGT>CGT (G12R) | wild type |
| 852 | F | 66 | 10 | Dead | PDAC | . | 3 | 3 | 1 | 0 | GGT>GAT (G12D) | wild type |
| 854 | M | 68 | 55 | Dead | PDAC | head region | 2 | 3 | 1 | 0 | GGT>GAT (G12D) | wild type |
| 856 | F | 42 | 1 | Alive | PDAC | body region | . | 3 | 1 | 1 | GGT>GAT (G12D) | wild type |
| 867 | F | 58 | 23 | Dead | PDAC | tail region | 2 | 3 | 1 | 0 | GGT>CGT (G12R) | deletion |
| ******  868 | M | 56 | 24 | Dead | PDAC | head region | 2 | 3 | 1 | 0 | GGT>GAT (G12D) | wild type |
| ****** 871 | F | 68 | 9 | Dead | PDAC | head region | 3 | 3 | 1 | 0 | GGT>GAT (G12D) | wild type |
| 944 | F | 69 | 18 | Alive | PDAC | head region | 2 | 3 | 1 | 0 | GGT>GAT (G12D) | CAC>TAC (H83Y) |
| 947 | F | 70 | 6 | Alive | PDAC | overlapping sites | 2 | 3 | 0 | 0 | wild type | wild type |
| 948 | M | 61 | 16 | Alive | PDAC | head region | 3 | 3 | 1 | 0 | wild type | deletion |
| 954 | F | 66 | 26 | Alive | PDAC | . | 3 | 3 | 1 | 0 | wild type | wild type |
| 971 | M | 67 | 7 | Alive | PDAC | head region | 2 | 3 | 1 | 0 | wild type | wild type |
| 995 | F | 61 | 22 | Dead | PDAC | head region | 2 | 3 | 1 | 0 | GGT>GAT (G12D) | wild type |
| 998 | F | 43 | 11 | Dead | PDAC | head region | 2 | 3 | 1 | 0 | GGT>GAT (G12D) | wild type |
| 1005 | F | 82 | 5 | Dead | PDAC | head region | 3 | 3 | 0 | 0 | GGT>GAT (G12D) | CGA>TGA (R58*) |
| 1006 | F | 76 | 22 | Dead | PDAC | head region | 3 | 3 | 0 | 0 | GGT>GAT (G12D) | wild type |
| 175 | M | 62 | 22 | Alive | SCA - benign | tail region | . | . | . | . | wild type | wild type |
| 391 | F | 71 | 9 | Alive | SCA - benign | tail region | . | . | . | . | GGT>TGT (G12C) | wild type |
| 692 | F | 24 | 3 | Alive | SCA - benign | head region | . | . | 0 | 0 | wild type | wild type |
| 964 | M | 71 | 16 | Alive | SCA - benign | tail region | . | . | 0 | 0 | wild type | wild type |
| 227 | F | 14 | 25 | Alive | SPN /Frantz' tumor | body region | . | . | . | . | wild type | wild type |
| 682 | F | 26 | 1 | Alive | SPN /Frantz' tumor | overlapping sites | . | . | . | . | wild type | wild type |

***** All patients received Gemcitabine as standard chemotherapy with exception of those who are shown to have received other treatments

****** Patients received 5FU/FA (5-fluorouracil/Folinic acid) treatment

******* Patient received 5FU and interferon alpha together with radiation therapy
